# Strategies involving low-molecular-weight heparin for the treatment and prevention of venous thromboembolism in patients with obesity: A systematic review and meta-analysis

**DOI:** 10.3389/fendo.2023.1084511

**Published:** 2023-03-08

**Authors:** Junjie Liu, Xi Qiao, Mingdong Wu, Haiyang Wang, Hailong Luo, Haolong Zhang, Yikuan Chen, Jianming Sun, Bo Tang

**Affiliations:** ^1^ Vascular, Abdominal & Hernia Surgery, The Second Affiliated Hospital of Chongqing Medical University, Chongqing, China; ^2^ Department of Clinical Medicine, The Second Clinical Medical College, Chongqing Medical University, Chongqing, China

**Keywords:** venous thromboembolism, obesity, anticoagulant, low-molecularweight heparin, treatment, prevention

## Abstract

**Introduction:**

Recent studies have indicated that the dosage of LMWH in patients with specific weights may be controversial. Therefore, we conducted a meta-analysis to explore an appropriate dosage of LMWH for the prevention and treatment of venous thromboembolism (VTE) in patients with obesity.

**Materials and methods:**

We searched the PubMed, EMBASE, and Cochrane Library databases up to July 23, 2022. Study selection, bias analysis, and information extraction were performed by three independent reviewers. The occurrence or recurrence of VTE and bleeding events were the primary outcomes we assessed.

**Results:**

Eleven studies (a total of 6266 patients) were included in the prevention group, and 6 studies (a total of 3225 patients) were included in the treatment group. For VTE prophylaxis, compared with the standard-dosage group, the high-dosage group had a lower incidence of VTE (OR: 0.47, 95% CI: 0.27-0.82, *P*=0.007) and a similar incidence of bleeding events (OR: 0.86, 95% CI: 0.69-1.08, *P*=0.020). For VTE therapy, compared to the standard-dosage group, the reduced-dosage group had a similar incidence of VTE recurrence (OR: 0.86, 95% CI: 0.11-6.84, *P*=0.89) but a lower incidence of bleeding events (OR: 0.30, 95% CI: 0.10-0.89, *P*=0.03).

**Conclusion:**

In patients with obesity, increasing the dosage of LMWH is a more appropriate option for the prevention of VTE. Due to the limited evidence, reducing the therapeutic dosage of LMWH requires careful consideration. Larger-scale, well-designed randomized controlled trials are necessary.

**Systematic Review Registration:**

https://www.crd.york.ac.uk/prospero/display_record.php?, identifier ID=CRD42022298128.

## 1 Introduction

Venous thromboembolism (VTE), including deep vein thrombosis (DVT) and pulmonary embolism (PE), is one of the major causes of in-hospital morbidity and mortality (1, 2). Obesity (BMI> 30 kg/m^2^) is an established strong and independent risk factor for the development and recurrence of VTE. The risk of VTE is estimated to be approximately 2.3 times higher in patients with obesity than in normal-weight patients (BMI 18.5-29.9 kg/m^2^) (3, 4).

Anticoagulation is the most crucial element in preventing and treating VTE. However, the strategies for anticoagulation in specific weight groups are still controversial. While several guidelines recommend the same agent and dosage as those given to normal-weight patients (5, 6), others do not address patients with specific weights (7, 8). Furthermore, according to the International Society on Thrombosis and Hemostasis (ISTH) guidelines, direct oral anticoagulants (DOACs) are not recommended for patients with a BMI>40 kg/m^2^ or a weight >120 kg (9).

Low-molecular-weight heparin (LMWH) is commonly used for the prophylaxis and treatment of VTE. Currently, patients with BMI>30 kg/m^2^ are often administered the same dosage as normal-weight patients. However, significant differences have been found in pharmacology between patients with obesity and normal-weight patients. After subcutaneous injection of a weight-dose (1.5 mg/kg) of enoxaparin, obese healthy volunteers had a higher level of anti-Xa exposure and a lower total body clearance than normal-weight volunteers (10). Furthermore, the lower volume of distribution in obese volunteers suggested that LMWH did not distribute into adipose tissue. Whether patients with BMI >30 kg/m^2^ need a dosage adjustment of LMWH is a topic of increasing concern in clinical decision-making.

Recent research shows that the use of a standard dosage of LMWH for prevention in this specific weight group may be inadequate (11–13). In a large-scale retrospective cohort study, despite receiving chemoprophylaxis, critically ill obese patients had a significantly higher incidence of VTE than nonobese patients (3). Moreover, for the treatment of obese VTE patients, some researchers have proposed a reduced dosage of LMWH, instead of 1 mg/kg twice daily (14, 15). Higher anti-Xa exposure at the same dosage may lead to a higher incidence of supratherapeutic anti-Xa levels. Research has shown a positive correlation between anti-Xa levels and BMI (15).

Thus, we performed a systematic review and meta-analysis to evaluate a higher dosage of LMWH for prophylaxis of VTE and a reduced dosage of LMWH for treatment of VTE in patients with obesity.

## 2 Methods and materials

In accordance with the Preferred Reporting Items for Systematic Reviews and Meta-Analyses (PRISMA) statement, this systematic review and meta-analysis was registered with PROSPERO as CRD42022298128.

### 2.1 Search strategy

PubMed, EMBASE, and the Cochrane Library databases were searched systematically and comprehensively for all available comparative studies from inception until July 23, 2022. The combination of search terms included “venous thromboembolism”, “low molecular weight heparin” and “obesity”. Additionally, we searched the references of the included studies for other relevant articles that were not found in the literature search. We present the complete search strategies for each database in **Supplementary Material (1)**.

### 2.2 Study selection

All the retrieved studies were imported into a citation manager (Endnote 20, Clarivate, Philadelphia USA). After removing duplicate studies, two authors (JJ Liu and X Qiao) systematically screened the remaining studies according to the titles, abstracts, and full text. Any disagreement was discussed in the case of a conflict, and a consensus was reached. Eligible studies met the following criteria for: (1) type of studies, namely, randomized controlled trials and high-quality cohort or case−controlled studies; (2) participants, namely, patients with BMI >30 kg/m^2^ needing prophylactic or therapeutic anticoagulation; (3) comparators, namely, use of a different dosage of LMWH; and (4) outcomes, namely, occurrence or recurrence of VTE, bleeding events, and anti-Xa levels.

Studies were excluded if they met the following criteria: (1) conference abstracts, letters, and comments; (2) studies not in English; (3) studies that involved patients who were pregnant or had renal impairment [creatinine clearance (CrCl)<30 ml/min]; and (4) studies with fewer than 10 participants in each group.

### 2.3 Definition of outcomes

The primary outcomes were the occurrence or recurrence of VTE, including PE or DVT, and the incidence of major and minor bleeding events during hospitalization or follow-up. Major bleeding was defined as a drop in hemoglobin of more than 2 g/dL, a transfusion of 2 or more units of blood products, or a retroperitoneal, intraocular, or intracranial hemorrhage. After major bleeding was ruled out, the remaining bleeding events were considered minor bleeding events (16).

The secondary outcomes were the incidence of supraprophylactic (supratherapeutic) anti-Xa levels and subprophylactic (subtherapeutic) anti-Xa levels. Although currently controversial (12, 17, 18), the prophylactic and therapeutic levels of anti-Xa are 0.2-0.4 IU/ml and 0.5-1.0 IU/ml, respectively.

### 2.4 Data extraction and risk of bias

We developed a predesigned data extraction sheet, and the data, including the study design, geographic location, sample size, intervention, and baseline data (i.e., age, BMI, sex, duration, renal function), were extracted independently by two coauthors (JJ Liu and MD Wu). Disagreements were resolved by discussion between the two review authors. If no agreement could be reached, a third author (B Tang) made the decision. Not all included studies monitored patients’ anti-Xa levels, and the characteristics of the enrolled studies that did not include patients’ anti-Xa levels were analyzed as a subgroup. Emails were sent for further information that was not provided in the full text.

The quality and risk of bias assessment of the studies included were independently evaluated by two coauthors (JJ Liu and MD Wu). The Cochrane Collaboration tool was used to assess the potential bias of randomized controlled trials. We used the Newcastle−Ottawa Scale (NOS) tool for cohort and case−controlled studies.

In addition, we used funnel plots and the Egger test to assess publication bias. A *P* value<0.05 indicated significant publication bias.

### 2.5 Statistical analysis

The occurrence or recurrence of VTE was used to evaluate the effectiveness of different dosages of LMWH for prophylaxis or treatment. The incidence of bleeding events was used for safety evaluation. As an adjunct, anti-Xa levels were included as another measure of efficacy if a small number of studies were included. The proportions and adjusted odds ratios (ORs) with 95% confidence intervals of the respective outcomes were calculated through meta-analysis. The inconsistency index (I^2^) statistics and the Q test were calculated for heterogeneity assessment. Low heterogeneity was defined as a *P* of *>* 0.1 and an I^2^ of <25%. When heterogeneity was low, a fixed-effects model was used; otherwise, a random-effects model was used. A *P* value < 0.05 was considered statistically significant. Subgroup analyses for the outcomes were performed according to the types of LMWH, bariatric surgery patients for prophylaxis, and supratherapeutic anti-Xa levels and subtherapeutic anti-Xa levels for treatment.

Review Manager v5.4 (Cochrane Collaboration, Copenhagen, Denmark), Stata v16.0 (StataCorp, Texas, USA), and R version 4.1.0 (R Foundation, 2021) were used for all of the statistical analyses.

## 3 Results

**Figure 1:**
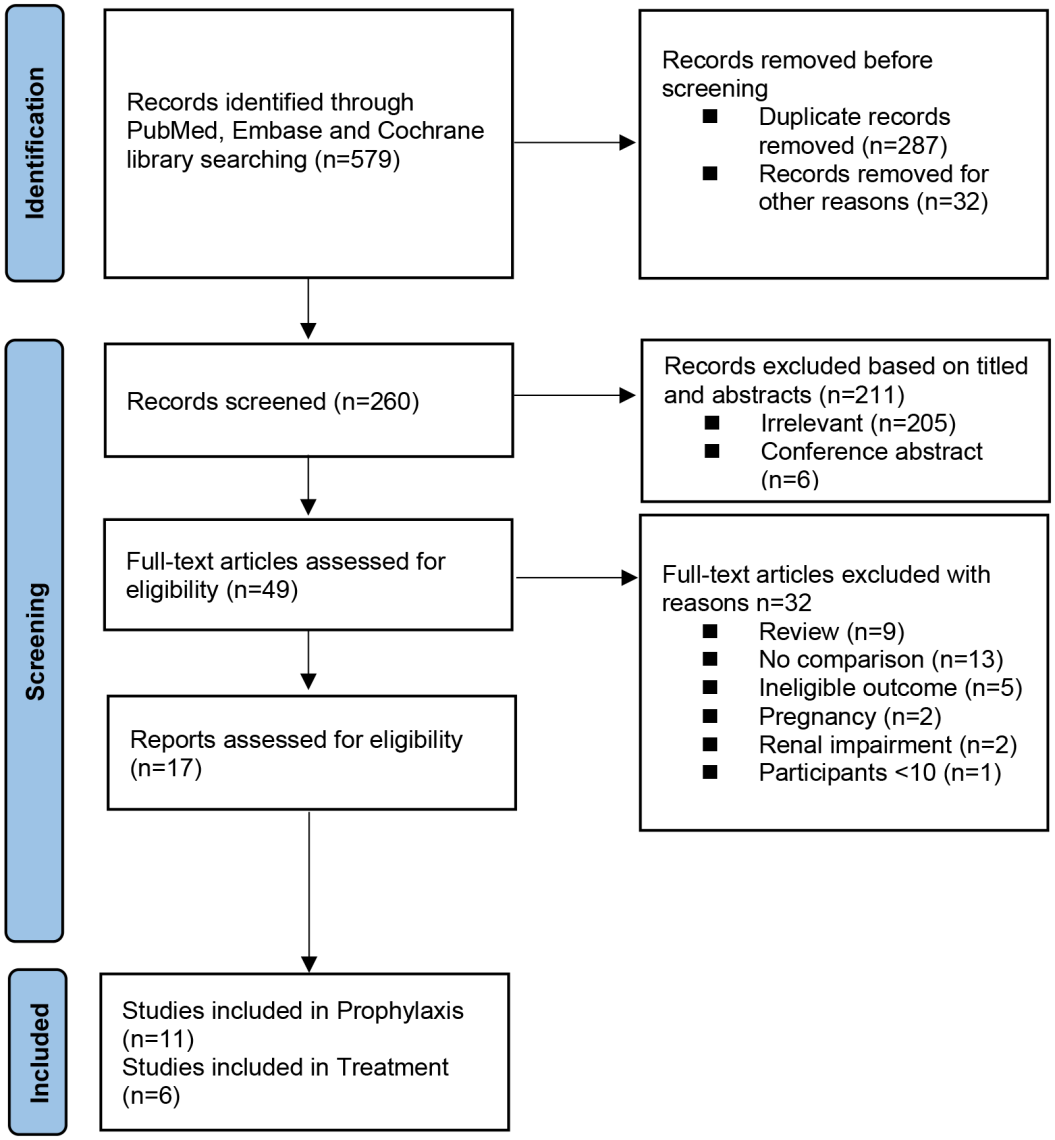
Flow diagram showing the progress of the included studies. The flow diagram template is derived from the open source template published in the 2020 version of the PRISMA guidelines, and is available for free download and use for all system reviews. https://guelphhumber.libguides.com/c.php?g=213266&p=1406923#:~:text=What%20is%20PRISMA%3F,a%204%2Dphase%20flow%20diagram.

The searches of the three databases provided 296 records. After adjustment for duplicates and other reasons, 168 studies remained. Of these, 113 studies were discarded because the papers did not appear to meet the criteria after review of the title and abstracts. Another 6 studies were excluded because they were conference abstracts. The full text of the remaining 49 citations was examined in detail. Thirty-two studies did not meet the inclusion criteria. Finally, a total of 17 studies involving 11 trials for prophylaxis (19–29) and 6 trials for treatment (30–35) were identified for inclusion in this meta-analysis. See the flow diagram in **Figure 1**.

The data on the number of patients who were administered LMWH could not be extracted from the full text of Wang TF (28) because a higher dosage of chemoprophylaxis consisting of unfractionated heparin (UFH) and LMWH was studied as a whole. An email was sent to obtain more detailed research data.

### 3.1 Characteristics of the included studies

#### 3.1.1 Prophylaxis

**Table 1** shows the baseline characteristics of the 11 included studies for prophylaxis. A total of 3153 participants with a standard dosage and 3113 with a higher dosage were included. These studies included 4 randomized controlled trials (19, 20, 23, 25), 5 prospective cohort studies (21, 22, 24, 26, 27), and 2 retrospective cohort studies (28, 29). Patients following bariatric surgery were the main target population for clinical VTE prevention (19–21, 24, 25, 27, 29). Of all the LMWH drugs, enoxaparin was most frequently provided for chemoprophylaxis in patients with BMI >30 (21, 23–29).

**Table 1:** Characteristics of the included prophylaxis studies.

Study	Design	Population	Agent	Dosage	Number of patients-n	Occurrence of VTE-n	Bleeding events-n	Suggest
Imberti (19) 2014	RCT	Bariatric	Parnaparin	S: 4250 IU/day H: 6400 IU/day	131 119	2 1	8 6	Standard
Kalfarentzos (20) 2001	RCT	Bariatric	Nadroparin	S: 0.6 ml/day H: 1.0 ml/day	30 30	0 0	0 2	Standard
Scholten (21) 2002	PCS	Bariatric	Enoxaparin	S: 30 mg/12 h H: 40 mg/12 h	92 389	5 2	1 1	Higher
Vavken (22) 2009	PCS	Orthopedic	Bemiparin	S: 3500 IU/day H: 5000 IU/day	83 667	1 2	0 0	Standard
Miranda (23) 2017	RCT	Medical inpatients	Enoxaparin	S: 40 mg/day H: 60 mg/day	45 46	0 0	3 2	Higher
Gelikas (24) 2017	PCS	Bariatric	Enoxaparin	S: 40 mg/day H: 60 mg/day	31 23	0 0	0 1	Controversial
Steib (25) 2015	RCT	Bariatric	Enoxaparin	S: 4000 IU/day H: 6000 IU/day	44 44	0 0	1 2	Higher
Gibson (26) 2021	PCS	Medical inpatients	Enoxaparin	S: 0.5 mg/kg/day H: 40 mg/12 h	40 40	0 0	0 0	Controversial
Simone (27) 2008	PCS	Bariatric	Enoxaparin	S: 40 mg/12 h H: 60 mg/12 h	24 16	0 0	1 0	Higher
Wang (28) 2013	RCS	Medical inpatients	Enoxaparin/UFH	S: 40 mg/day enoxaparin 5000 IU/8or12h UFH H: 40 mg/12h enoxaparin 7500 IU/8 h UFH	2369 1559	35 12	200 112	Higher
Hamad (29) 2005	RCS	Bariatric	Enoxaparin	S: 40 mg/day H: 60 mg/12 h	264 180	0 2	3 3	Controversial

S, Standard dose. H, Higher dose

VTE, Venous thromboembolism. RCT, Randomized controlled trial. PCS, Prospective cohort study. RCS, Retrospective cohort study. USA, United States of America. UFH, Unfractionated heparin.

As in normal-weight patients, most studies used subcutaneous 40 mg daily of enoxaparin as the standard dosage and 60 mg daily or 30 mg twice daily as the higher dosage (23–25, 27–29). Few studies used a weight-based dosage (0.5 mg/kg^2^) as a prophylactic option (26). It is worth noting that the standard dosage of the prophylaxis group at a few institutions was higher (40 mg twice daily) (27). To explore whether the inclusion would affect the research conclusion, we conducted a sensitivity analysis.

Dosage selection for the remainder of the studies was performed using site-specific criteria according to different types of LMWH drugs and different medical centers. Five studies recommended a higher dosage as a prophylactic strategy in patients with obesity (21, 23, 25, 27, 28), 3 studies maintained the traditional strategy (19, 20, 22), and an additional 3 studies considered the current findings controversial because the studies were small scale and the evidence was insufficient (24, 26, 29).

#### 3.1.2 Treatment

**Table 2** shows the baseline characteristics of the 6 treatment studies, including 1 randomized controlled trial (32), 1 prospective cohort study (31), and 4 retrospective cohort studies (30, 33–35). A total of 3225 participants were included in this group (2616 with a standard dosage vs. 609 with a reduced dosage). Enoxaparin remained the preferred type of LMWH for patients with BMI >30 (31–33, 35). A weight-based dosage of 1.0 mg/kg q12h was commonly used as a standard dosage, and a reduced dosage of LMWH referred to less than 1.0 mg/kg, which was approximately 0.8 mg/kg (30–32, 35). Almost all studies recommended dosage reduction for treating VTE in patients with BMI>30 (30–33, 35), except for one retrospective cohort study that used dalteparin (34).

**Table 2:** Characteristics of the included treatment studies.

Study	Design	Population	Agent	Dosage	Number of patients-n	Recurrence of VTE-n	Bleeding events-n	Suggest
Mirza (30) 2020	RCS	VTE	LMWH	S: 20865 IU/day R: 18000 IU/day	2392 454	6 2	24 0	Reduced dose
Thompson-Moore (31) 2015	PCS	VTE/AF/ CAHD	Enoxaparin	S: 1.0 mg/kg/12 h R: 0.83 mg/kg/12 h	18 23	0 0	4 4	Reduced dose
Curry32 2018	RCT	VTE/AF	Enoxaparin	S: 1.0 mg/kg/12 h R: 0.8 mg/kg/12 h	26 28	0 0	0 0	Reduced dose
Van Oosterom (33) 2019	RCS	VTE	Enoxaparin	S: >0.85 mg/kg/12 h R: <0.85 mg/kg/12 h	67 66	2 0	2 0	Reduced dose
Smith (34) 2003	RCS	VTE/AF/ CAHD	Dalteparin	S: 126.2 units/kg/12 h R: 196.5 units/kg/day	11 10	0 0	0 0	Controversial
Maclachlan (35) 2019	RCS	VTE	Enoxaparin	S: 1.0 mg/kg/12 h R: <1.0 mg/kg/12 h	102 28	0 0	4 0	Reduced dose

S, Standard dosage. R, Reduced dosage

VTE, Venous thromboembolism. AF, Atrial fibrillation. CAHD, Coronary atherosclerotic heart disease

RCT, Randomized controlled trial. PCS, Prospective cohort study. RCS, Retrospective cohort study. USA, United States of America.

### 3.2 Bias assessment

For the quality and potential bias assessment of RCTs, selection bias, performance bias, detection bias, attrition bias, and reporting bias were evaluated with the Cochrane Collaboration tool, and no disagreements occurred between the two coauthors. The NOS tool was used for cohort studies. We assessed a total of 8 items in terms of selection, comparability, and outcome and scored each study with a total score of 9. A score of less than 6 was considered a high risk of bias study. All bias assessment results are presented in **Supplementary Material (2)**.

### 3.3 Meta-analysis of the included studies

In the meta-analysis, we excluded studies with no outcome events occurring in either the experimental group or the control group. Ultimately, 5 studies for efficacy assessment and 9 studies for safety assessment in the VTE prevention group were enrolled. Only 2 studies for efficacy assessment and 4 articles for safety assessment in the treatment group were included. Considering that few studies were included to evaluate the effectiveness of the reduced dosage of LMWH, we included anti-Xa levels in the effectiveness evaluation and compared the incidence of subtherapeutic anti-Xa levels. The higher the incidence was of not reaching anti-Xa therapeutic levels, the lower the efficacy. A therapeutic level referred to 0.5-1.0 IU/ml. The anti-Xa levels of the included studies are presented in **Supplementary Material (3)**. A meta-analysis was performed for each level group, and 4 studies were included in each subgroup.

#### 3.3.1 Prophylaxis

**Figure 2:**
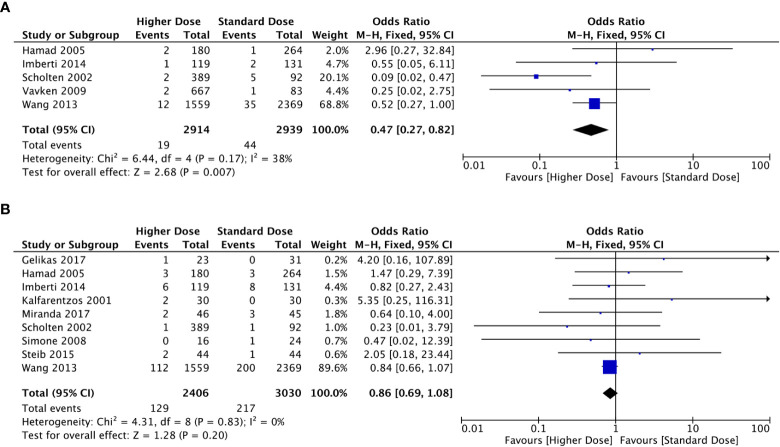
Forest plots involving higher dosage versus standard dosage of LMWH for the prophylaxis of VTE in obese patients. **(A)** occurrence of VTE; **(B)** bleeding events.

The results of the meta-analysis in the prophylaxis group are presented in **Figure 2**. Overall, increasing the dosage of LMWH tended to decrease the incidence of VTE events. Combining all studies, the incidence of VTE was approximately 0.65% (n=2914) in the higher-dosage group, which increased to 1.50% (n=2939) in the standard-dosage group (OR: 0.47, 95% CI: 0.27-0.82, *P*=0.007). In addition, the heterogeneity test suggested that the heterogeneity between studies was low (*P*=0.17; I^2 =^ 38%).

Bleeding events occurred in 129 of 2406 patients in the higher-dosage group and 217 of 3030 in the standard-dosage group. A higher dosage of LMWH did not significantly increase the risk of bleeding in patients with obesity, and the incidence of bleeding events was similar between the two dosages (OR: 0.86, 95% CI: 0.69-1.08, *P*=0.020). No significant heterogeneity was observed in the meta-analysis (*P*=0.83; I^2 =^ 0%).

#### 3.3.2 Treatment

**Figure 3:**
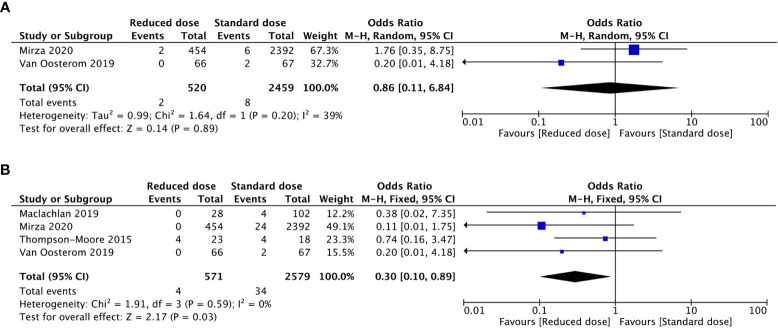
Forest plots involving reduced dosage versus standard dosage of LMWH for the treatment of VTE in obese patients. **(A)** recurrence of VTE; **(B)** bleeding events.

**Figure 4:**
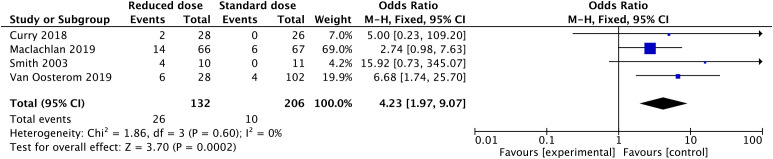
Forest plots involving reduced dosage versus standard dosage of LMWH for the treatment of VTE in obese patients: patients below the therapeutic anti-Xa level;.

The recurrence of VTE after anticoagulant treatment was low. Only 10 obese patients in two included studies experienced recurrent VTE (2 of 520 with a reduced dosage vs. 8 of 2459 with a standard dosage). After a meta-analysis of the two studies, there was no significant difference in the incidence of recurrent VTE between the reduced-dosage group and the standard-dosage group (OR: 0.86, 95% CI: 0.11-6.84, *P*=0.89) (**Figure 3**). Considering the bias caused by the small number of included studies, we performed a meta-analysis of rates of subtherapeutic anti-Xa levels in the included studies (**Figure 4**). Compared with the standard-dosage group, the proportion of patients in the reduced-dosage group who did not reach the therapeutic level was significantly higher (OR: 4.23, 95% CI: 1.97-9.07, *P*=0.0002). No significant heterogeneity was observed.

After an overall meta-analysis of the incidence of bleeding events between the reduced-dosage group and the standard-dosage group, the use of a reduced dosage in obese patients was significantly associated with a lower incidence of bleeding events (OR: 0.30, 95% CI: 0.10-0.89, *P*=0.03). No significant heterogeneity was observed among the 4 included studies (*P*=0.59; I^2 =^ 0%).

### 3.4 Subgroup, sensitivity analysis, and publication bias

**Figure 5:**
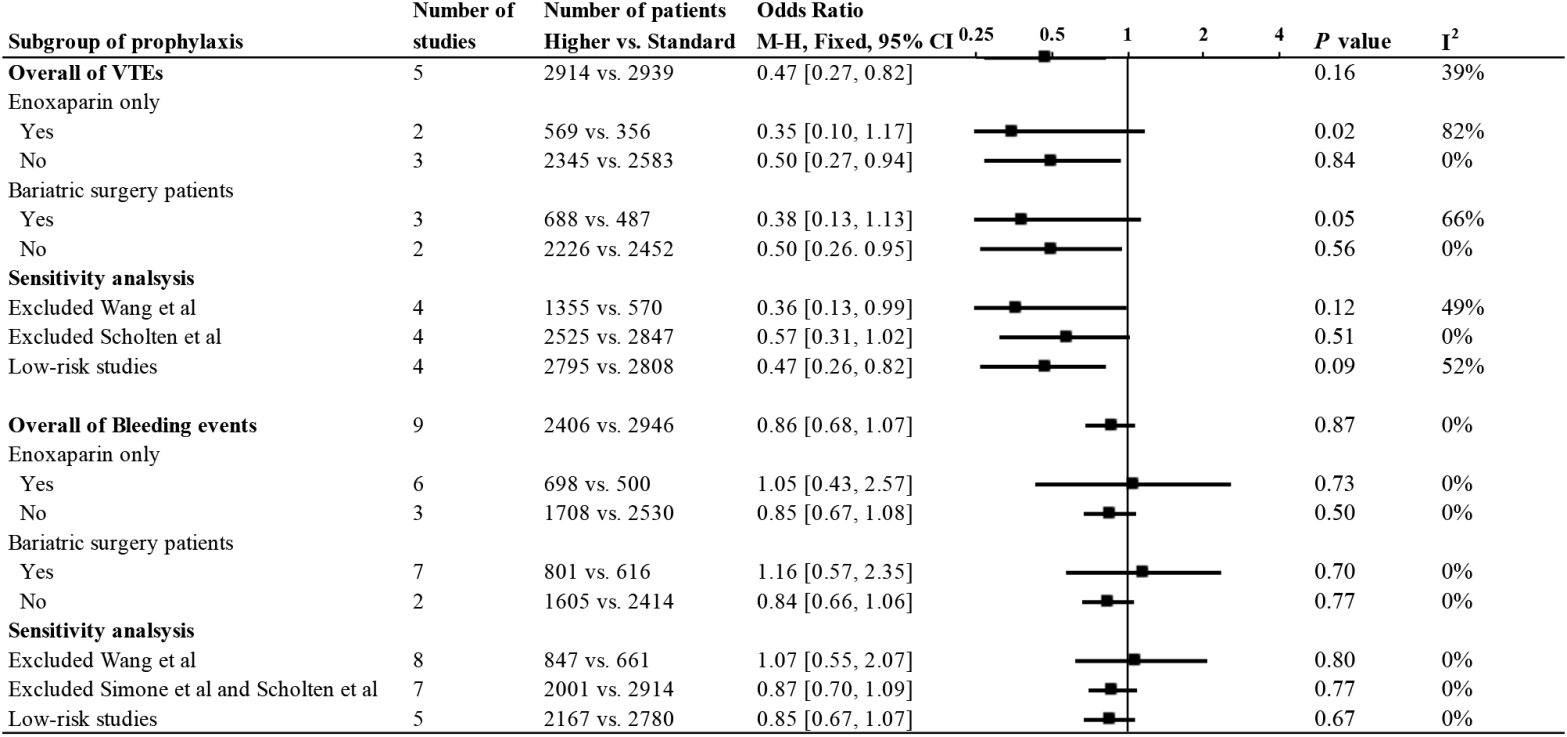
Subgroup analysis and sensitivity analysis.

We performed a subgroup analysis of the meta-analysis of prophylaxis in obese patients (**Figure 5**). Groups were divided according to the use of enoxaparin only and bariatric surgery patients, and the efficacy and safety were both analyzed. The results for each subgroup were basically consistent with the overall statistical results.

Sensitivity analysis excluded the study by Wang et al. because UFH was incorporated in this study. The results of the meta-analysis after this exclusion were consistent with the main results, and no heterogeneity was found in the efficacy and safety analysis. In addition, we performed another sensitivity analysis of the remaining low-risk studies after excluding higher-risk studies based on the quality bias analysis of each study. The quality of the included studies did not significantly affect the conclusions of the studies.

We performed the Egger test for several major studies, including the efficacy and safety of the prophylactic group and the safety of the therapeutic group, with respective Egger test values ​​of P=0.894, 0.485, and 0.097. No publication bias was found. Funnel plots are presented in **Supplementary Material (4)**.

## 4 Discussion

The use of LMWH for anticoagulation in patients with BMI > 30 kg/m^2^ is controversial. There is the possibility of insufficient anticoagulation with a standard dosage. Moreover, an aggressive dosing strategy will cause excessive anticoagulation and increase the bleeding risk. Pharmacological findings also suggest that our research is necessary. To the best of our knowledge, this is the first meta-analysis to evaluate LMWH strategies in patients in this specific weight group.

In this meta-analysis, 11 studies were included in the prevention group, and 6 studies were included in the treatment group. The final statistical analysis of the prophylaxis group found that a higher dosage of LMWH reduced the incidence of VTE without increasing the risk of bleeding. In the treatment group, a reduced dosage was associated with a reduced incidence of bleeding events. After combining the two studies on the efficacy of LMWH treatment, we found that the outcome was favorable for reducing the dosage in obese patients. When we attempted to address this issue by including anti-Xa levels in the efficacy evaluation, the results were contradictory. A reduced dosage of LMWH failed to provide adequate anti-Xa levels in patients with obesity. Due to the limited number of studies, whether to reduce the therapeutic dosage of LMWH in obese patients needs further verification.

In a large retrospective cohort study, Wang et al. combined LMWH and UFH to observe the effect of high-dosage chemoprophylaxis on anticoagulation in obese patients (28). However, the full text of the article does not mention the statistical data for enoxaparin. We learned by email from the authors that 69% of obese patients were administered enoxaparin, while another 31% were administered UFH. Considering the large number of patients included in this study and the similarities in the pharmacology of LMWH and UFH, this article was still included in our study by a unanimous decision of the three investigators. To explore the impact of this study on the final results, we conducted a sensitivity analysis. After the study was removed, the statistical results were still consistent with the main statistical results.

A standard prophylactic dosage of LMWH in most institutions is 40 mg QD, and higher dosages of LMWH are total dosages of 60-80 mg daily (19, 20, 22–26, 28, 29). We included two additional studies using 30 mg BID and 40 mg BID as standard dosages and 40 mg BID and 60 mg BID as higher dosages. The two studies ultimately supported the higher dosage recommendation as well. This suggests that an increased dosage of prophylaxis is warranted; however, a total dosage of 60 mg daily may not be optimal. Further research is needed to explore optimal prophylactic dosages for patients in this specific weight group. A sensitivity analysis of the two included studies was conducted, and after excluding them, the results remained consistent.

Gibson et al. compared the difference between a weight-based dosage (0.5 mg/kg) and a fixed dosage (40 mg twice daily) (26). There was no significant difference in anti-Xa levels between the two regimens. However, due to the small size, no outcome events occurred. Larger-scale studies are therefore needed to investigate the applicability of weight-based versus fixed dosages in patients with BMI>30.

There was no clear reduced dosage as a treatment option for obese patients, and 0.8 mg/kg q12h appeared to be the choice in some institutions (31, 32). However, due to the low incidence of recurrent VTE and the limited number of included studies, only two studies had recurrent VTE events. After a meta-analysis of the two studies, the results were in favor of reducing the dosage in obese patients. To address this, we introduced anti-Xa factor levels. Anti-Xa levels below the lower limit of the therapeutic standard (0.5 IU/ml) were considered inadequate. Notably, the results indicated that the dosage of the reduced group was significantly insufficient.

Therefore, the current evidence does not directly demonstrate that reducing the therapeutic dosage in patients with obesity can achieve the same effect. Even the incidence of bleeding events was significantly reduced. We still have doubts about whether reduced dosages would result in insufficient anticoagulation. Therefore, conclusions should be considered with caution until further research takes place.

We performed subgroup analysis and sensitivity analysis only on the meta-analysis of prophylaxis. Owing to the fact that the number of studies included in the meta-analysis of the treatment group was small. This showed that the conclusions of the treatment group needed to be treated with more caution. In subgroup analyses, we discussed the effect of enoxaparin and bariatric surgery on the study findings. Effectiveness analysis showed that although the grouping created heterogeneity, the forest plot showed a trend that did not be changed. We attribute this to the small number of included studies. In addition, in the safety analysis, subgroup and sensitivity analysis were consistent with the final conclusion, and there was no statistical heterogeneity. Due to the limitation of the number of research studies, we performed publication bias testing on the efficacy and safety of studies in the prevention group and the safety of studies in the treatment group, and the results were negative.

The need for anti-Xa monitoring in obese patients is another controversial topic. Routinely, some studies did not recommend anti-Xa monitoring unless the patient was at significant risk of major bleeding, especially for prophylaxis (17, 35–37). Several other studies suggested that anti-Xa monitoring in obese patients is necessary to make dosage adjustments (32, 38). In this meta-analysis, after combining the included studies, the incidence of VTE was 0.65% with a higher dosage for prophylaxis and 0.38% with a reduced dosage for treatment. Therefore, given the low incidence of VTE, anti-Xa monitoring is not recommended for obese patients unless further studies demonstrate a benefit.

Our study excluded obese patients with atrial fibrillation. The anticoagulant strategy for patients with atrial fibrillation is mainly based on out-of-hospital oral anticoagulants (warfarin and DOACs), and LMWH is mainly used as bridging anticoagulation in the perioperative period of patients with atrial fibrillation (39). In addition, related research was limited. It showed that this may be an overlooked area and further research is needed to explore perioperative anticoagulation strategies in this particular population.

Furthermore, since our study focused on hospitalized obese patients administrated LMWH, the use of DOACs in obese patients was not included in our research. DOACs in obese patients also face challenges. Relevant systematic studies and meta-analyses had shown that the use of DOACs in obese patients was safe, and the efficacy of DOACs in various weight groups might not be affected by body fat (40, 41). In addition, DOACs could reduce the risk of bleeding in obese patients compared with warfarin. DOACs may be a safe and effective option for out-of-hospital obese patients.

This systematic review and meta-analysis has several limitations. First, although randomized controlled trials were included in both the prevention and treatment sections, they included small sample sizes. Large-scale studies are still being conducted retrospectively. This may have an unpredictable effect on our statistical analysis. Because of the instability of dosage maintenance in retrospective studies, grouping by dosage is not strictly controlled. Second, due to individual differences in drug types and clinical centers, not all studies had the same standard dosages and altered dosages. Such dosage differences across studies, especially in the prevention component of meta-analyses, may affect the incidence of outcomes. Third, the number of studies included in the reduced dosage efficacy analysis for treatment was smaller due to the low recurrence rate of VTE. Even though we assessed anti-Xa levels as a supplementary analysis, the reference treatment level of anti-Xa was not clearly defined. Due to the different therapeutic levels of anti-Xa, we analyzed the subtherapeutic group and the supratherapeutic group to ensure the combination ability of each study group, but it did not reflect the real effectiveness of the reduced dosage in obese patients. Further large-scale studies are needed to verify the efficacy. Fourth, although we included studies in obese patients with BMI >30, in fact, the BMI values of obese patients in each study varied from 30 to 60, so the analysis of drug dosages for patients with a higher BMI is not accurate. Unfortunately, due to the lack of baseline data, a subgroup analysis of BMI values could not be performed in this study.

## 5 Conclusion

Our systematic review and meta-analysis show that compared with the standard dosage, a higher dosage of LMWH to prevent VTE in patients with obesity can reduce the incidence of VTE without increasing the risk of bleeding. Due to limited evidence, the option of reducing the therapeutic dosage should remain cautious until further studies are available. Larger-scale, well-designed randomized controlled trials are necessary.

## Data availability statement

The original contributions presented in the study are included in the article/**Supplementary Material**. Further inquiries can be directed to the corresponding author.

## Author contributions

JL: study design, literature retrieval, data collection, data analysis and manuscript writing. XQ: study design, data collection, data analysis and manuscript writing. MW: literature review, data collection and manuscript writing. HW: literature retrieval, data analysis, and manuscript writing. HL: study design and manuscript revision. HZ: conception and manuscript revision. JS: data analysis, manuscript revision. YC: data analysis, manuscript revision. BT: conception, manuscript revision All authors contributed to the article and approved the submitted version.
