# Supplementary material for: Strategies involving low-molecular-weight heparin for the treatment and prevention of venous thromboembolism in patients with obesity: A systematic review and meta-analysis
Source: Front Endocrinol (Lausanne). 2023 Mar 8;14:1084511. doi: 10.3389/fendo.2023.1084511 (PMC10031025; doi:10.3389/fendo.2023.1084511)
Supplement: Supplementary file 1 [file DataSheet_1.docx]

1. **Search strategies in each database**
   1. PubMed

Search terms: (venous thromboembolism or deep venous thrombosis or pulmonary embolism [Title/Abstract]) AND (obesity or overweight [Title/Abstract])) AND (low molecular weight heparin or dalteparin or enoxaparin or nadroparin or tinzaparin[Title/Abstract])

Results: 394

- 1. Embase

Search terms: obesity: ab, ti AND 'low molecular weight heparin': ab, ti AND 'venous thromboembolism'/dm

Results: 157

- 1. The Cochrane Library

Search terms: "low molecular weight heparin" in Title Abstract Keyword AND obesity in Title Abstract Keyword AND venous thromboembolism in Title Abstract Keyword - (Word variations have been searched)

Results: 28

- 1. From included studies

Results: 0

1. **Bias assessment of included studies**
   1. Randomized controlled trials

Figure 2.1. Risk of bias bar chart of combined studies

Figure 2.2. Risk of bias domains in each RCT.

- 1. Cohort studies

Table 2.1 Results of quality assessment using the Newcastle‒Ottawa Scale for case‒control studies

| Study | Selection | Comparability | Exposure/Outcome | Scores |
| --- | --- | --- | --- | --- |
| Scholten  2002 | *** | * | ** | 6 |
| Vavken  2009 | **** | * | * | 6 |
| Gelikes  2017 | **** | ** | ** | 8 |
| Gibson  2021 | **** | * | * | 6 |
| Simone  2008 | *** | * | * | 5 |
| Wang  2013 | *** | ** | * | 6 |
| Hamad  2005 | ** | * | * | 6 |
| Mirza  2020 | *** | * | ** | 6 |
| Thompson-Moore  2015 | *** | * | ** | 6 |
| Van Oosterom  2019 | *** | * | ** | 6 |
| Smith  2003 | ** | * | *** | 6 |
| Maclachlan  2019 | *** | ** | *** | 8 |

1. **Anti-Xa levels in included treatment studies**

Table 3.1. Anti-Xa levels in included treatment studies.

| Study | Reference  Anti-Xa levels (IU/ml) | Number of patients (n) | Mean anti-Xa  (IU/ml) | Number of subtherapeutic levels (n) |
| --- | --- | --- | --- | --- |
| Curry  2018 | 0.5-1.1 | S:26  R:28 | 0.86  0.80 | 0  2 |
| Van Oosterom  2019 | 0.5-1.0 | S:67  R:66 | NA  NA | 6  14 |
| Smith  2003 | 0.5-1.0 | S:11  R:10 | 0.90  1.10 | 0  4 |
| Maclachlan  2019 | 0.5-1.0 | S:102  R:28 | 0.93  0.77 | 4  6 |

*S:* Standard dose*. R:* Reduced dose *NA:* Not Applicable

1. **Publication bias funnel plots**

Figure 4.1. Funnel plot for publication bias test on the incidence of VTE in prophylaxis (P=0.894)

Figure 4.2. Funnel plot for publication bias test on the incidence of bleeding in prophylaxis (P=0.485)

Figure 4.3. Funnel plot for publication bias test on the incidence of bleeding in treatment (P=0.097)
